# Supplementary material for: Are carbon emissions trading and green financial instruments synergistic? -Comprehensive quantitative research based on content analysis
Source: PLoS One. 2024 Mar 7;19(3):e0298601. doi: 10.1371/journal.pone.0298601 (PMC10919601; doi:10.1371/journal.pone.0298601)
Supplement: S1 Data — (ZIP) [file pone.0298601.s001.zip › Data/地级市Z_F工作报告.zip!/╔┬╬≈/╬╝─╧╩╨/└┤╫╘╣1⁄2╓┌║┼í╛┬φ┐╦╩2╛▌═°í┐_2014.docx]

各位代表：

　　现在，我代表市人民政府向大会作工作报告，请予审议，并请各位政协委员和列席会议的同志提出意见。

　　一、五次会议确定任务完成情况

　　刚刚过去的一年，是我们面对困难稳中求进、陕西东大门建设取得显著成绩的一年。一年来，在市委的正确领导下，市政府团结依靠全市人民，认真贯彻落实党的十八大和十八届三中全会精神，开拓创新，真抓实干，全市经济社会继续保持平稳较快发展。生产总值完成1349亿元，增长12%；财政总收入114.7亿元，增长 8.5%，其中地方财政收入65亿元，增长18.1%；城镇居民人均可支配收入和农民人均纯收入分别达到24164元和7565元，增长10.8%和14.6 %；社会消费品零售总额达到367.2亿元，增长15.3%。

　　（一）在抢抓机遇中持续扩大投资，发展动力显著增强。260个市级重点项目完成投资1246亿元，占年度计划的103.8%，50个项目建成投产，36个前期项目提前开工，澄城烟厂技改、西石化搬迁等前期工作取得突破性进展。成功举办了首届黄河金三角投资合作交流大会，全年签约招商引资项目337个，到位资金494.5亿元，增长20.3％。全社会固定资产投资完成1467.6亿元，增长25.2%。

　　（二）在平稳增长中推进产业升级，转型发展步伐加快。现代农业稳步发展。粮食总产211.2万吨，省级现代农业园区发展到28个，陕西现代畜牧业示范区建设进展顺利，“大荔模式”成为全国农资农技双服务的典范，富平成为全省唯一的国家现代农业示范区改革与建设试点示范县。工业经济平稳增长。全市规模以上工业实现总产值1728.5亿元、增加值603.3亿元。高新区国家级新材料高新技术产业化基地获得科技部批准，陕煤70万吨聚烯烃生产线项目进入设备安装阶段，投资60亿元的陕化节能技改项目建成投产，投资50亿元的经开区申华汽车博展园项目开工建设。新增规模以上工业企业72户，工业园区建成区面积达到110.9平方公里，入园企业达到924户。第三产业快速发展。出台了《关于促进大华山旅游目的地建设的若干意见》，华阴市率先开通旅游免费公交，潼关成为全国第一个景区无门票县市，华山景区网络媒体形象评价在全国5A景区中排名第一，全市接待游客2900万人次，实现旅游综合收入212亿元，增长36.3%。新培育发展限上贸易企业95户，第三产业实现增加值403.4亿元，增长9.6%。金融机构存款余额1522.4亿元，贷款余额726.02亿元，分别增长13%和16.6%。

　　（三）在加快城镇化中推进美丽渭南建设，城乡面貌深刻变化。中心城市实施建设项目163个,完成投资163.8亿元。城市“五纵六横一环”道路网络基本形成，供、排水管网覆盖率分别达到90%和84%。渭河综合整治堤防工程基本完成，城区段2万亩河滩全面实施退耕还河还湿地,中心城市“一河两岸”发展格局初现雏形。10个县城建成区面积达到176平方公里，城区人口达到148万人。5个省级重点示范镇完成投资11.1亿元,2个省级文化古镇和12个市级重点镇建设全面启动，建成城市社区168个、农村社区971个。16.8万农民进城落户，城镇化率达到38.13%。坚持将节约的“三公”经费用于环境治理，彻底取缔了富平、蒲城土法石灰窑。秦东大地园林化工程成效明显,完成造林绿化54.8万亩。“治污降霾·保卫蓝天”行动扎实推进,节能减排各项指标均实现省考目标，市区空气质量达标天数309天。

　　(四)在推动科学发展中着力改善民生，人民群众得到更多实惠。全年财政民生支出224.8亿元，占全市财政支出的81.5%。新中心医院建成投用，市体育中心、市文化艺术中心和市博物馆基本建成；实施韦罗高速、国道108改造等交通项目40个，完成投资30.1亿元；筹资1802万元，为816户失独家庭发放了一次性补助金；城乡居民社会养老保险基础养老金提高到每人每月70元；新增城镇就业7.4万人，劳动力转移就业130.3万人，城镇登记失业率控制在3.3%；65岁以上老人免费健康体检40.3万人，4.5万农村孕妇免费住院分娩，34.4万70岁以上老人享受津贴，15万人享受医疗和临时救助，新农合参合率和城镇基本医疗覆盖面均达到99%；建成各类保障房18918套；34万城乡低保人口应保尽保，18.5万人实现脱贫，33.2万人饮水安全得到解决；渭南职教集团组建顺利，合阳县“双高双普”正式通过省级评估验收，“学前教育三年行动计划”圆满完成；成功承办了2013赛季全国男子篮球联赛(NBL)等重大赛事，渭南籍运动员武柳希在全国第十二届运动会上摘取金牌；公共文化服务“四进”零距工程获第十届“中国文化艺术节”项目类群星奖，华阴老腔亮相联合国教科文组织“中国日”活动和2014中央新春团拜会；安全生产事故起数和死亡人数同比下降56.48%和68.16%，信访“三无”创建和平安渭南建设深入推进，公众安全感进一步提升，社会大局和谐稳定。

　　（五）在解放思想中推进改革开放，发展空间进一步拓宽。食品安全监管体制改革走在全国前列，企业工商登记制度改革在全省率先启动，“营改增”试点进展顺利，组建了4个国有企业集团，完成了12户商贸企业改制。韩城计划单列试点进展顺利，简政放权取得阶段性成效，下放县市区经济管理权限127项，向社会中介组织转移职能49项。行政事业单位分类改革初见成效，减少财政供养人员553名。对外开放不断扩大,渭南海关建设进展顺利,与西安市政府签订了《西渭融合发展战略合作框架协议》，与韩国龟尾市开展了友好合作交流互访,与黄河金三角等城市的联系日益密切。坚持科技引领、人才支撑，与12家高校院所签定战略合作协议，全市科技研发投入13.23亿元，技术交易额完成6094万元,专利授权量529件。首次从优秀工人农民中考试录用公务员27人,先后引进博硕士人才132名,柔性引进院士、专家75名。

　　（六）在转变作风中加强自身建设，政府的执行力和公信力明显提升。省级依法行政示范市创建扎实推进，重大决策群众听证、专家咨询等制度有效落实。自觉接受市人大及其常委会的法律监督和市政协的民主监督，完善报告工作和联系沟通机制，人大代表建议和政协委员提案办复率达到100%。严格执行中央八项规定，深入开展群众路线教育实践活动调研工作，扎实开展纪律作风大整顿再排查和“当好标杆，向我看齐”、电视问政、“向人民报告”等活动。全市“三公”经费下降10.06%，会议费下降26.29%，有力促进了政府系统作风转变和效能提高。

　　同时，国防动员、双拥工作、国家安全、人民防空、民族宗教、外事侨务不断加强，妇女、儿童、老龄、残疾人、红十字事业全面进步，监察、审计、统计、气象、档案、地方志、新闻出版和广播电视等各项工作都取得了新的成绩。

　　各位代表，一年来，在严峻挑战和困难较大的情况下，全市经济社会发展总体好于预期。主要指标增速均高于全省平均水平，部分指标位居全省前列；食品药品改革、公共文化服务、环保、地震、体育等多项工作受到国家表彰，群众满意度较上年提高了两个百分点；我市入选全国资源成熟型城市序列，并被确定为国家智慧城市建设试点和公共文化服务体系创建示范区；中心城市成功创建省级文明城市，蒲城、合阳、潼关和大荔进入国家卫生县城行列，韩城、富平荣获“全国科技进步考核先进县”；以反“四风”为重点的干部作风建设扎实推进，成为各级干部干事创业新的动力。这些成绩的取得，是市委正确领导的结果，是市人大、市政协和社会各界监督支持的结果，是全市人民团结奋斗的结果。在此,我谨代表市人民政府,向全市广大干部群众、驻渭部队指战员、武警官兵和公安干警致以崇高的敬意！向大力支持政府工作的各位代表、各位委员和各界人士表示衷心的感谢！

　　在肯定成绩的同时，我们也清醒地看到，我市仍处于欠发达地区行列，经济总量小，结构不够合理，城镇化水平、经济外向度和居民收入低的问题依然突出；去年经济增长低于预期目标1.5个百分点，财政收入占GDP的比重相对较低；资源环境约束日益趋紧，投资持续高速增长难度增加；市场主体培育还需进一步加强，改善民生与群众的期待仍有差距；影响社会和谐稳定的因素依然较多，安全事故仍时有发生；少数政府工作人员的作风群众还不满意，特别是个别负面典型案件的发生，影响了渭南的对外形象，伤害了渭南人民的感情。对此，我们将采取有效措施切实加以解决，绝不辜负人民群众的期望。

　　二、2014年主要工作目标和任务

　　2014年，是贯彻落实十八届三中全会精神、全面深化改革的开局之年，也是实施“十二五”规划、加快建设陕西东大门的攻坚之年。虽然当前经济发展中的不利因素依然较多，我市发展仍然面临着扩充总量和提升质量的双重压力。但国家、我省经济社会发展基本面长期向好，仍处于重要的战略机遇期。我市是人口大市，区位优越，基础资源条件好，城镇化水平低但潜力巨大，经过多年努力，各县市区都具备了向更高层次发展的条件。同时，中央全面深化改革、加快推进新型城镇化、共建丝绸之路经济带等战略部署也为我们带来了新的重大机遇。我们一定要抢抓机遇，创造机会，努力把各项工作做得更好，使渭南发展的更好更快，让群众得到更多实惠。

　　综合考虑，今年全市经济社会发展的主要预期目标是：生产总值增长12%以上，地方财政收入增长10%，全社会固定资产投资增长25%，社会消费品零售总额增长16%，城乡居民收入增长12%和13%，城镇登记失业率控制在4%以内，人口自然增长率控制在6‰以内，万元GDP能耗下降3.8%。

　　实现上述目标，一定要牢牢把握稳中求进的工作总基调，好中求快，提质增效，形成更加科学的发展思路；一定要坚持项目带动，强化投资支撑，打造更加突出的发展优势；一定要深化改革、推进创新，不断消除体制机制障碍，催生更加强劲的发展活力；一定要注重以人为本、改善民生，倾情倾力为群众办实事、做好事、解难题，凝聚更加强大的发展力量，为把渭南打造成面向大中原、融入大西安的关天开放之门、宜居宜业的生态田园新城、产业结构优化升级的东西合作高地、规模化品牌化标准化市场化的现代农业基地和国际一流的旅游胜地而不懈努力。

　　（一）以抓项目扩投资为抓手，强化经济发展支撑

　　坚持把扩大投资作为加快发展的主要抓手，力争全社会固定资产投资达到1835亿元。

　　集中推进重大项目。坚持领导包抓、讲评观摩等措施，全力加快280个市级重点项目建设，确保年度完成投资1200亿元。做好环境保障，支持西气东输三线、神渭输煤管线、大西铁路、宁西铁路复线、渭玉高速等中省项目加快建设。确保黄河雨林上延续建工程主汛期前完工，阳山庄钢铁炉料、富平生态水泥上半年投产，陕煤70万吨烯烃、青岛啤酒搬迁一期工程三季度建成，加快蒲白高速等交通重点工程建设，韦罗高速和连霍高速渭南西出入口改造年内竣工。力促澄城烟厂技改、富平热电联产、禹门口黄河大桥等年内开工建设，西石化搬迁年内完成项目选址及可研阶段任务。

　　重视项目策划争取。准确把握国家投资重点和产业导向，紧盯汽车类项目、化工能源加工项目、资源类深加工项目等，精心策划重大项目特别是产业投资项目，确保年内策划重大项目200个以上。完善大项目组建班子、小项目确定专人的工作机制，最大限度地争取中省政策和资金支持。

　　切实加大招商力度。紧紧围绕优势资源开发、产业链条延伸、工业园区发展、高新技术引进等，大力开展招商引资。积极开展央企对接，承接产业转移，加快引进战略投资者。注重以商引商，强化驻外办事机构的招商职能，完善项目跟进和落地服务机制，确保招商引资到位资金达到560亿元，利用外资5000万美元。

　　着力优化投资环境。坚持把投资环境作为生命线工程来抓。确保市行政服务中心上半年建成投用。全面推行服务承诺、限时办结等制度，探索推行“负面清单”制度。健全完善制度体系，靠制度管人，用制度管事。牢固树立“企业家功高、纳税人至上”的理念，下大力气解决企业和群众反映强烈的突出问题，坚决查处破坏投资环境的典型案件，确保投资环境从根本上得到改善。

　　（二）以产业转型升级为引领，加快推进新型工业化

　　以高端化、集约化、效益化为方向，着力推进转型发展，提升产业层次，增强工业竞争实力。

　　推进产业转型升级。突出发展新兴产业，加快国家级新材料高新技术产业化基地和增材制造（3D打印）培育基地建设,努力将渭南打造成增材制造（3D打印）产业的中试站、孵化器、聚集地。积极支持拓日新能、LED光电产业等稳产促销，不断提高战略性新兴产业的比重。加快传统产业改造升级，着力推动能源化工、有色冶金、装备制造、食品加工等产业提档升级。抓住列为全国资源成熟型城市的有利机遇,增强资源型产业可持续发展能力。高度重视建筑业发展,加快培育一批竞争力强的优势建筑企业,推动房地产市场健康发展,提高建筑业对经济发展的贡献。

　　培育壮大企业群体。继续落实财政奖励等扶持政策，促进龙钢、金钼、中联重科、黑猫焦化、陕化等重点骨干企业释放产能、提质增效。鼓励引导优势企业通过重组、并购等方式扩大规模，扶持成长潜力大、发展好的企业做大做强，年内新增规模以上工业企业50家。

　　大力发展非公经济。落实扶持政策，放宽市场准入，激发民间投资活力。完善中小企业公共服务平台功能，加快中小企业孵化基地建设，推动民营经济快速发展。鼓励外出成功人士回乡创业，积极推进全民创业，让更多的劳动者成为创业者。

　　做强产业发展平台。按照产城融合理念，加快工业园区标准厂房、公租房和服务平台建设，完善功能配套，提升承载能力。落实支持高新区、经开区加快发展若干意见，开展园区托管模式试点，着力提升县市工业园区发展水平。加大龙头企业引进力度，强化上下游配套和产品开发，推动链条化、集群化发展，力争园区工业增加值增长20%以上。

　　强化科技和人才支撑。组建市科技资源统筹中心。整合相关资金，支持重点企业与科研院所联合建立技术中心或研发机构，申报中省高新技术产业化专项，推动科技创新成果产业化，使渭南成为全省科技成果转化的承载地。力争全社会科技研发投入占GDP的比重达到1%以上，建成市级工程技术研究中心10个。积极实施人才强市战略，大力发展职业教育，加快培养高素质技能型人才和产业工人，着力引进高层次人才、紧缺人才和重点人才，不断壮大领军人才队伍和创新创业团队。

　　（三）以人为核心推进城镇化，打造生态田园新城

　　坚持以人为本、生态宜居、规划引领、产业支撑、集约智能、绿色低碳，切实加快新型城镇化步伐。

　　促进城乡规划全覆盖。编制全市新型城镇化规划。搞好渭南新区规划和主城区综合管网、城市风貌等专项规划编制，进一步完善医院、学校等社会事业布局，力争中心城市规划覆盖率达到100%。完成韩城和华阴城市总体规划、建制镇总体规划修编和新型农村社区规划编制工作。坚持以人为本、尊重自然、天人合一的理念，着力提高规划水平，让城市延续历史、融入自然，使居民望得见山、看得见水、记得住乡愁。

　　提高中心城市首位度。实施中心城市建设项目190个，完成投资266亿元。以强化基础、聚集产业、搞活商业、提升人气为重点，着力提高高新区发展层次，打造以高新技术产业为标志的国家级高新区形象；集中支持经开区加快发展，注重产城融合，突出田园都市特质，打造渭南新区；加快渭河城区段堤顶道路和景观建设，推进河滩退耕还河还湿地工程和两岸开发建设，基本建成滨河景观长廊，使渭河真正成为城中河、生态河、景观河；大力支持临渭区实施老城区开发，抓好规划编制，加快改造步伐，恢复历史风貌。同时，进一步完善城市基础设施和功能配套，打通解放路、前进路北段等城市道路，加快北水厂建设，3月底前启动抽黄供水工程建设，力争到2015年底，使城市供水问题从根本上得到解决。实施城市“温暖”工程，加大城中村和棚户区改造力度，推进城市精细化管理，启动国家文明城市创建工作，全面加快百万人口大城市建设步伐。

　　加快县城和小城镇发展。以扩容提质、一城三创为重点，提高县城标准化建设水平。突出抓好19个省市重点镇和文化旅游古镇建设。加快重点镇“扩权强镇”步伐，赋予重点镇更大的经济社会管理权限，打造县城副中心。以14个省市新型农村示范社区为带动，加快新型农村社区建设，使农民不离乡土就能享受城市生活。

　　推进农业转移人口市民化。深化户籍制度改革，完善教育、医疗、社保和住房等保障机制，推动农村居民进城落户，年内转移户籍人口12.5万人。把进城落户人员纳入城镇住房保障、社会保障和公共服务体系，着力解决城乡医疗养老保险整合、随迁子女入学等问题，努力让进城落户人员平等享受市民待遇。

　　（四）以增加群众收入为重点，提升现代农业发展水平

　　坚持以规模化提高产量、以标准化提高品质、以品牌化提高声誉、以市场化提高效益，着力推进西部现代农业先行区建设，加快现代农业发展步伐。力争到2020年，农业总产值达到600亿元，农产品加工和流通业产值超过1500亿元。

　　强化粮食安全意识。严守耕地红线，优化品种结构，加强灌区改造和农田水利基础设施建设，加快建设高标准粮食生产集中区，确保粮食总产稳定在210万吨以上。

　　做大做强特色产业。积极实施果业提质增效工程，建设现代果业强市，力争全市水果面积达到300万亩以上。以设施栽培为重点，推进“菜篮子”工程提档升级，力争全市设施蔬菜面积达到100万亩以上。进一步加快陕西现代畜牧业示范区建设，确保畜牧业产值增长10%以上。

　　实施农业品牌战略。实施区域公用品牌建设，整合提升“白水苹果”、“蒲城酥梨”、“韩城花椒”、“大荔冬枣”、“临渭葡萄”等区域品牌。加强对名优农产品的产地保护、商标注册和广告宣传，加快建立无公害、绿色、有机和地理标志认证农产品生产基地。制定综合标准，建立农产品质量追溯体系，探索发展智慧农业，力争年内创建省级以上名牌产品8个、市级名牌产品12个。

　　推进农业产业化发展。积极探索土地入股等多种方式，推进土地流转，引导各类社会资本参与园区建设、经营和管理，加快万亩农业园区建设，力争年内新增市级以上园区15个，园区总面积达到70万亩。注重向农业的二产和三产要效益，使农业不仅成为富民产业，更成为强市产业。着力扶持各类农产品加工企业发展壮大，年内新增市级龙头企业20户以上。提升农业科技服务水平，实现“大荔模式”乡镇全覆盖。

　　（五）以打造国际一流旅游胜地为目标，大力发展第三产业

　　把发展第三产业作为转变发展方式的战略重点，力争第三产业增加值达到458亿元，增长12%。

　　突出发展文化旅游业。整体推进大华山旅游目的地建设，加快华山申遗步伐，实施西峰索道及310国道沿线景观提升工程，加快“太华湖”生态旅游度假示范区等项目建设，推进洽川、少华山、潼关古城等景区提档升级。加快沿黄公路二期工程建设，力争渭河大桥、洛河大桥年内开工，促进沿黄旅游开发。加快韩城“风追司马”、 富平红色旅游、蒲城唐陵和白水林皋国际慢城等旅游开发。推进文化与旅游融合发展，深化与黄河金三角城市的旅游协作。实施旅游标准化战略，丰富旅游产品，提升服务水平，努力让游客在渭南慢下来、住下来、乐起来。力争全年接待游客3200万人次，旅游综合收入达到252亿元。

　　大力发展现代服务业。编制全市商业网点规划和物流业发展规划，加快万达城市综合体、红星美凯龙、经开区申华汽车博展园、潼关物流港等项目建设。加强商贸流通服务体系建设，着力引进高端商贸企业来渭发展，积极发展各类服务业，全年新增限额以上贸易企业80户。进一步优化金融环境，积极支持商业银行等在渭南设立分支机构，规范担保公司和小额贷款公司，打击非法融资，年内新增各类贷款150亿元。

　　着力扩大市场消费。扶持文化旅游消费，稳步发展住房和汽车等大宗消费，加快培育网络购物等消费热点。推进中心城市特色商业街区规划建设，延时公交运营，延长商业网点营业时间，不断激活市场消费。继续搞好双百市场、蔬菜进社区、放心粮油全覆盖等工程。力争全年社会消费品零售总额达到426亿元。

　　(六)严守生态红线，打好生态治理和环境保护攻坚战

　　牢固树立生态红线观念，建立健全科学考评机制，加强环境治理与保护，建设美丽渭南。

　　加强生态建设。组织实施南塬城区段全面绿化，持续推进秦东大地园林化，完成植树造林35万亩。巩固小流域治理成果，治理水土流失350平方公里。加快渭河综合整治，完成二华蓄滞洪区主体工程。全面实施卤阳湖湿地生态恢复工程，抓好国家湿地公园申报，围绕湿地积极发展高科技和生态旅游产业，打造关中“绿肺”。扎实推进生态县、生态镇和生态村创建，力争华县、华阴创建为省级生态县。

　　狠抓环境治理。尽管去年我们取缔土法石灰窑、关闭秦岭开山采石、整治烟花爆竹等，影响了1个百分点左右的GDP，但环境治理的决心不能动摇。强力落实“治污降霾·保卫蓝天”行动计划，进一步改善空气质量。持续推进渭河三年治污行动，确保年底实现“三年变清”目标。坚决淘汰落后产能，扎实推进重点减排项目建设，确保实现节能减排各项目标。通过“关小上大”、引进高新技术、发展替代产业等，促进建材产业做优做强。

　　加快美丽乡村建设。借鉴白水、大荔、合阳等县经验，狠抓农村清洁工程和垃圾处理，推进农村环境连片综合治理，根本改善农村环境面貌，实现美丽与发展双赢。

　　（七）突出重点守住底线，持续加大保障和改善民生力度

　　改善民生是政府的首要责任。我们将按照保基本、补短板、兜底线的要求，坚持把新增财力和财政支出“两个80%”用于民生，扎实落实各项民生措施，努力使群众生活的更加幸福、更有尊严。

　　千方百计扩大就业。全面落实就业创业政策，加强技能培训和就业服务，力争城镇新增就业6.2万人，农村劳动力转移就业100万人以上。

　　提高社会保障水平。做好农民工、非公企业职工、灵活就业人员等群体社会保险扩面提标工作，推进社会保障由补缺型向普惠型转变。加强医保基金征缴，探索推行大病统筹。开工建设保障房4.7万套，推行保障房源、分配过程、分配结果“三公开”制度，着力提高群众满意率。加强社会救助和城乡低保管理，推进社会化养老服务体系建设。

　　加大扶贫开发力度。实行专业扶贫、精准扶贫，启动秦岭北麓和渭北旱塬扶贫移民搬迁七年行动计划，年内搬迁0.8万人。抓好208个扶贫开发整村推进项目建设。着力推进连片扶贫开发，年内实现18万人脱贫。

　　坚持教育优先发展。强力推进“双高双普”县创建，继续实施千校达标工程。确保渭南初级中学、经开区渭南中学9月1日建成开学。启动第二期“学前教育三年行动”，新建改建幼儿园31所。整合重组全市职业教育资源,推动渭南职教集团快速发展。积极鼓励发展民办教育。着力优化教师资源配置，促进义务教育均衡发展。提升教育质量，强化教育安全，办好人民满意教育。

　　加强公共卫生工作。启动建设渭南市儿童医院。鼓励社会资本兴办医疗机构，推动民营医院进入城镇居民医疗保险和新合疗范围。巩固完善基本药物制度，推进基本公共卫生服务均等化。统筹解决人口问题，提高出生人口素质。

　　积极发展社会事业。扎实搞好第三次经济普查。广泛开展全民健身运动，精心办好市第十二届运动会。扎实推进国家公共文化服务体系示范区创建，年内建成市图书馆，做好“两馆一站”免费开放规范化服务，抓好重点镇综合文体中心建设和村级文化活动器材配送，支持优秀剧目和精品力作，促进城乡文化大发展、大繁荣。

　　着力维护安全稳定。坚持对安全生产隐患实行“零容忍”，推进烟花爆竹行业专项整治，强化煤炭等重点行业安全监管，严格责任追究，坚决杜绝重特大事故发生。加强基层基础工作，逐步改善镇村和社区办公条件。深入开展双拥共建活动，认真做好民族宗教工作，精心搞好第九次村委会换届选举。加强社会治理创新，注重以法治的思维、法治的方式处理信访问题，妥善化解各种不稳定因素，扎实推进平安渭南建设，严厉打击各类刑事犯罪，确保全市社会大局稳定。

　　（八）深化改革推进开放，不断激发发展活力

　　改革是最大的红利。我们将认真贯彻党的十八届三中全会和省委、市委的部署，把握大局，主动作为，胆子要大，步子要稳，确保改革取得实效。

　　深化行政管理体制改革。稳步推进市县政府机构改革。加大支持韩城计划单列试点力度，加快华县改县设区步伐。深入推进简政放权，加快事业单位分类改革。开展党政机关和事业单位控编减编工作，力争年内财政供养人员减少1300人。

　　加快资源配置市场化改革。大力发展混合所有制经济，强化市场在资源配置中的决定性作用。加快工商登记制度改革，加大市场主体培育力度。坚持政企分开，推动城投公司成为融资平台和盈利中心。积极推进城乡建设用地增减挂钩和工矿废弃地复垦利用试点，深化农村综合改革，加快农村集体土地确权登记工作。

　　加强财税金融改革。推进市以下财政体制改革，完善重点镇财政体制。县级以上部门全部公开部门预决算和“三公”经费。开展跨部门、跨项目、跨类别整合资金试点，建立财政支出绩效评价机制。推进“营改增”试点和税收一体化管理。鼓励民间资本参与地方金融机构改革，分层次实施全市农信社向农村商业银行改革。充分利用资本市场，推动有条件企业上市融资。

　　推进社会事业改革。深化教育、卫生综合改革，启动实施“单独二孩”政策。进一步完善深化县镇基层食品药品全程监管机制，确保食品药品安全。推进事业单位养老保险试点和城乡居民医保一体化试点。完善物价补贴联动机制，建立低收入行业工资正常增长机制。

　　不断扩大对外开放。推进西渭融合发展，引导西安产业向渭南有序转移，吸引西安及周边群众来渭休闲度假、观光旅游。利用西安三星效应，吸引韩国企业来渭投资。组织筹办第二届黄河金三角投资合作交流会，进一步加强与黄河金三角城市的经济协作，积极参与丝绸之路经济带建设，确保渭南海关年内开关运行。在开放合作、主动作为中，使渭南的发展空间不断拓宽。

　　三、进一步加强政府自身建设

　　全面深化改革，加快转型发展，对政府工作提出了新的更高要求。我们一定要以强烈的使命意识和责任意识，全面加强政府作风建设、法制建设、廉政建设和勤政建设，不断提高政府的公信力和执行力。

　　以群众路线教育实践活动为载体，持续推动作风转变。深入群众“接地气”，体察民情“聚民智”，坚定不移反“四风”。立行真改、从我做起，当好标杆、向我看齐，切实密切干群关系。牢固树立“为政不勤也是腐败”的观念，着力改进文风会风，严肃整治不作为、慢作为和乱作为，大力弘扬“低调务实不张扬、埋头苦干争一流”作风，努力将教育实践活动的成效体现在经济社会发展上，转化为惠民利民的实际成果。

　　以建设法治政府为目标，提升依法行政水平。深入开展“六五”普法宣传，坚持集体学法制度，提高依法决策能力。规范行政执法行为，强化执法监督，增强政府公信力。自觉接受市人大及其常委会的法律监督和市政协的民主监督，高质量办理人大代表建议和政协委员提案。坚持科学民主决策，落实公众听证、专家论证、风险评估、合法性审查和集体讨论决定等程序，保证各项重大决策符合人民意愿，经得起实践和历史检验。

　　以创新服务管理为支撑，提高公共服务能力。办好“12345”政府呼叫服务中心，健全基层便民服务体系，推动实现县有便民服务大厅、镇有便民服务中心、村有便民服务室。办好政务微博，扩大政务公开。继续深入开展“向人民报告”、 网络问政、电视问政等活动，不断畅通联系群众的渠道。

　　以源头治腐厉行节约为重点，切实加强廉政建设。严格贯彻落实中央八项规定和厉行节约反对浪费条例，确保“三公”经费在上年基础上压缩5%以上。全面推进惩治和预防腐败体系建设，强化行政问责、审计监督和效能监察。对各类腐败行为坚决实行零容忍，对政府工作人员违规违纪坚持实施顶格处理，让人民群众实实在在感受到政府作风在一天天好转。

　　各位代表，军政军民团结是巩固发展我市大好局面的重要保证。长期以来，驻渭部队和武警官兵为渭南发展作出了重要贡献。我们将一如既往地支持部队建设，注重对军事设施的保护，深入开展国防教育，加快推进军民融合，健全国防动员体系，搞好预备役部队和民兵建设，不断开创双拥共建工作新局面。

　　各位代表，科学发展任重道远，美好前景催人奋进。让我们在市委的坚强领导下，凝心聚力、埋头苦干，奋发进取、激情创业，切实加快陕西东大门建设步伐，共同谱写全市人民美好生活的新篇章！
